# A Letter to the Right Honorable Sir Henry Hardinge, K.C.B. M.P. on the Effects of Solitary Confinement on the Health of Soldiers, in Warm Climates

**Published:** 1837-07

**Authors:** 


Art. II.—*A Letter to the Right Honorable* Sir Henry Hardinge, k.c.b. m.p., *on the Effects of Solitary Confinement on the Health of Soldiers, in Warm Climates.* By John Grant Malcolmson, f.r.a.s. and m.g.s., Surgeon E. I. C. Service; late Secretary Madras Medical Board.—*London,* 1837. 8vo. pp. 23.

We have always been of opinion that strict solitary confinement, and also what has been called the silent system, would be found detrimental to health of mind, body, and affections. Governments, yet unskilled in the means of securing the general good behaviour of communities, are perpetually harassed with the necessity of inventing punishments, none of which have yet been devised which answer the sole useful or justifiable ends of punishments,—the deterring of the bad from crime, and the consequent protection of the good. The civil and the military authority is here equally impotent. Imprison a man who lives by robbing, and you secure him the comforts he chiefly wants in the winter season, without the necessity of exercising his troublesome and dangerous vocation. The treadmill, to be sure, is an unpopular exercise, and grinding corn by the hand is unpleasant; but these labours are the lot of lesser criminals, dull rogues who are only beginning their career: he knows nothing of either. He would be better pleased if you allowed him spirits and cigars; but he has food, clothing, and shelter, and, when the fine weather comes, he will be at liberty. Transport a felon, and he thanks you most sincerely: he goes to a better climate. If you hang him, he is personally more inconvenienced; but his friends cheer him at the foot of the scaffold, and it is doubtful whether the execution makes even one rogue the less. Then the soldier, flog him, and you spoil him for life, and at the expense of the hatred of the whole regiment; shut him up in a cell, and he turns sulky, loses his appetite, declines his bread, and introduces the scurvy to the care of the staff-surgeon. There is, we take it, a very plain lesson in all these failures, but men are not yet prepared to read it.

In the mean time, the plan of placing a poor devil in a situation in which all the stimuli of life are withdrawn from him does not answer. His stomach will not digest; his heart will not circulate blood freely; his brain will not secrete nervous power; the very tissues of his bodily frame become disordered, and break up. In a warm climate, these results are still more certain. "I have reason to believe," says Mr. Malcolmson, "that more real misery has arisen in twelve months from imprisonment in the great jails of India, than has been inflicted by corporal punishment in a hundred years:" and he gives a statement shewing that between thirty and sixty per cent. was the mortality in a few of these institutions in 1833 and 1834.

A strong impression exists in our minds, that whatever corps presents frequent instances of punishment is badly commanded. We think Mr. Malcolmson's experience causes him to entertain the same opinion: he mentions that he has witnessed the good effects of a better system in one European corps in India, "in which, while every man was required strictly to perform his duty, the gates of the barracks were left open, and none of those annoying restrictions, once so common, were imposed. Under this system, the men were remarkably healthy; only one instance of corporal punishment occurred in several years, and solitary confinement was seldom called for."

Until April, 1832, European prisoners in India were allowed their ordinary rations, excepting spirits. General orders were then issued, restricting the diet to bread and water, with such addition as a medical officer might deem necessary. The bad effects of this alteration soon became manifest, and they are clearly represented by Mr. Malcolmson in the following passage:

"I shall make no apology for subjoining a very brief outline of the effects observed to follow protracted solitary confinement, more especially where the prisoner is restricted to a diet of bread and water: reserving minute medical details for another place.

"Many men, particularly those of indolent habits, endure a confinement of four or six weeks, on bread and water, without injury to their health; but in some instances a shorter period is sufficient to cause a total loss of appetite; the bread is hardly touched, and, on other food being allowed, the patient is unable to eat or to digest it. The stomach becomes weak; there is uneasiness across the region of the stomach, spleen, and liver; the latter is torpid; the bowels are confined, or they are relaxed with slimy discharges, unaccompanied with pain, yet the swollen red tongue indicates the existence of irritation of the mucous membrane of the digestive canal. The pulse is quick and feeble; and the clammy skin, vertigo, debility, headach, and sleeplessness shew how much the constitution suffers from diminished nervous power. The convalescence is slow, and the treatment requires to be adapted to the enfeebled state of the system. The effect is, however, more clearly seen in men sentenced to six or twelve months' solitary confinement. Two of these were in hospital at the same time, with decided symptoms of scurvy: one was admitted after five months' confinement, during part of which he had been allowed extra diet, at my recommendation. It was observed that, for some time previous to his removal to hospital, his daily allowance of bread was removed almost untouched. He complained of pains of the limbs, along the spine, and across the loins; tenderness of the shin-bones; hardness, pain, and feeling of stiffness of the calves of the legs, and the skin over the painful muscles was of a dark livid colour, from effused blood. The gums were spongy, livid, and retracted; and he suffered from sleeplessness, some pain of the region of the liver, and slight griping. The tongue was yellow, and its edges red.—The other had been a shorter time in confinement, and complained of debility, disorder of the bowels, pains of the shin-bones, &c. A blister was applied, which caused a foul sore, from which dark-coloured blood flowed on the slightest touch. My friend, Mr. James Shaw, having furnished me with a report on the health of these men, two years after I left the regiment, I am enabled to state, that the one had hardly been out of hospital during that time, and had not then completed his full period of confinement; and that the other was very frequently on the sick list with a variety of complaints. Indeed, very few men are able to undergo a long period of solitary confinement on bread and water, without being much in hospital during the period of sentence; and many continue to suffer from the various diseases to which men of exhausted constitutions are so liable in warm climates. It may not be improper to add, that I have observed the minds also of prisoners confined for long periods, more especially when on a diet they believe to be destructive to their health, to become gloomy, or even furious, and disposed to commit every crime; a fact which was forcibly stated, many years ago, in the House of Commons, by Sir Robert Peel, in reference to the substitution of solitary confinement for other punishments in this country. When the solitary confinement is long continued, the severity of the punishment is increased in a much greater proportion than the length of time, and any addition in the shape of restricted diet, which may be necessary in short confinements, is quite uncalled for; the long seclusion without employment is itself sufficient." (P. 12.)

This letter is evidently the production of a clear-headed and sensible man, and is calculated to do much good: we shall endeavour to make its contents known where they are likely to be appreciated with effect.

